# Reconstruction of dual-frequency conductivity by optimization of phase map in MREIT and MREPT

**DOI:** 10.1186/1475-925X-13-24

**Published:** 2014-03-08

**Authors:** Oh In Kwon, Woo Chul Jeong, Saurav Z K Sajib, Hyung Joong Kim, Eung Je Woo, Tong In Oh

**Affiliations:** 1 Department of Mathematics, Konkuk University, 143-701 Seoul, Korea; 2 Department of Biomedical Engineering and Impedance Imaging Research Center, Kyung Hee University, 446-701 Yongin, Korea

**Keywords:** MRI, MREIT, MREPT, Conductivity, Magnetic flux density, Optimization

## Abstract

**Background:**

The spectroscopic conductivity distribution of tissue can help to explain physiological and pathological status. Dual frequency conductivity imaging by combining Magnetic Resonance Electrical Property Tomography (MREPT) and Magnetic Resonance Electrical Impedance Tomography (MREIT) has been recently proposed. MREIT can provide internal conductivity distributions at low frequency (below 1 kHz) induced by an external injecting current. While MREPT can provide conductivity at the Larmor frequency related to the strength of the magnetic field. Despite this potential to describe the membrane properties using spectral information, MREPT and MREIT techniques currently suffer from weak signals and noise amplification as they both reply on differentiation of measured phase data.

**Methods:**

We proposed a method to optimize the measured phase signal by finding weighting factors according to the echo signal for MREPT and MREIT using the ICNE (Injected current nonlinear encoding) multi-echo pulse sequence. Our target weights are chosen to minimize the measured noise. The noise standard deviations were precisely analyzed for the optimally weighted magnetic flux density and the phase term of the positive-rotating magnetic field. To enhance the quality of dual-frequency conductivity images, we applied the denoising method based on the reaction-diffusion equation with the estimated noise standard deviations. A real experiment was performed with a hollow cylindrical object made of thin insulating film with holes to control the apparent conductivity using ion mobility and an agarose gel cylinder wrapped in an insulating film without holes to show different spectroscopic conductivities.

**Results:**

The ability to image different conductivity characteristics in MREPT and MREIT from a single MR scan was shown by including the two objects with different spectroscopic conductivities. Using the six echo signals, we computed the optimized weighting factors for each echo. The qualities of conductivity images for MREPT and MREIT were improved by optimization of the phase map. The proposed method effectively reduced the random noise artifacts for both MREIT and MREPT.

**Conclusion:**

We enhanced the dual conductivity images using the optimally weighted magnetic flux density and the phase term of positive-rotating magnetic field based on the analysis of the noise standard deviations and applying the optimization and denoising methods.

## Background

The conductivity spectra of biological tissues can provide diagnostic medical information from the estimation of physiological and pathological conditions of *in-vivo* and *ex-vivo* tissue. However it is difficult to produce high resolution conductivity images inside the human body [1,2]. The conventional conductivity imaging methods have limited spatial resolution and sensitivity inherited from the ill-posed nature of the problem [3]. In order to achieve sensitive conductivity images with high resolution, electric impedance imaging techniques based on a magnetic resonance imaging (MRI) have been propsed. These include magnetic resonance electrical impedance tomography (MREIT) and magnetic resonance electrical property tomography (MREPT) which are under active investigation [4-17]. Both methods use the internal magnetic field information obtained from the phase data of MRI scanner to reconstruct the internal conductivity image.

MREPT can provide the electrical conductivity information at the Larmor frequency by measuring the phase of positive rotating field due to the applied *B*_1_ field. It does not require application of external current and therefore directly recovers the conductivity distribution by taking the derivative of the measured phase signals twice. MREIT needs a pair of electrodes to inject current into an imaging object during MRI scan for measuring a magnetic flux density induced by the external injecting current. The MREIT technique used only the *z*-component of magnetic flux density, *B*_*z*_, of **B**=(*B*_*x*_,*B*_*y*_,*B*_*z*_) to reconstruct the cross-sectional apparent conductivity image at a lower frequency range (below 1 kHz) [18-22]. The phase difference approach with an interleaved encoding scheme was adopted to cancel the systematic artifacts accumulated in phase signals and also reduce the random noise artifacts. Recently, a simultaneous conductivity imaging technique using a combination of MREPT and MREIT was proposed to provide the dual-frequency conductivities of tissue from a common MR scan [23]. MREPT using *B*_1_-mapping technique visualizes the conductivity and permittivity distributions at the Larmor frequency and MREIT recovers the apparent conductivity distribution when injecting low-frequency external current through the attached electrodes. Since the biological tissues show the frequency dependent conductivity property [1,2], the simultaneous dual-frequency conductivity imaging using a single MR scan is beneficial to provide distinct electrical features of tissues quantitatively.

Both conductivity imaging techniques use the phase signals of measured MR data. MREIT and MREPT commonly suffer from weak signals and noise amplification by using the derivative of the measured signal. The noise level of *B*_*z*_ in MREIT is inversely proportional to the signal-to-noise ratio (*Υ*^*j*^) of the MR magnitude image and the current injection pulse width. Because of the small amount of injection current and the poor quality of measured *B*_*z*_ change due to the injected current, it is difficult to perform *in vivo* human experiments using a conventional MR pulse sequence. To enhance the magnetic flux density due to the injected current in MREIT, the injected current nonlinear encoding (ICNE) method was introduced. This extended the duration of injecting current until the end of a readout gradient, and improved the signal by using a multi-echo train MR pulse sequence [24-26]. MREPT also suffers from low sensitivity due to the inherently poor signal to noise ratio and noise sensitive characteristics as it also needs the derivative of measured data, is very sensitive to the measured noise.

In this paper, we adopt a multiple spin echo MREIT pulse sequence based on the ICNE scheme to measure multiple phase data for MREIT and MREPT images. The acquired multiple phase data can be decomposed to the phase term reflecting the magnetic flux density signal induced by the injected current and the other phase term of positive rotating field due to the applied *B*_1_ field. We analyze the noise level of the two decomposed phase terms and minimize the measured random noise artifacts by applying the optimal combination of multiple phase terms. Also, we apply a denoising technique to the optimized magnetic flux density for MREIT and to the phase signal for MREPT in order to improve the quality of the reconstructed conductivity images. We prepared a conductivity phantom consisted of two different kinds of anomalies to show the difference of MREIT and MREPT. A phantom experiment is conducted to validate that the proposed method is able to improve the qualities of reconstructed dual-frequency conductivity images compared to the results of using conventional MREIT and MREPT reconstruction algorithms.

## Methods

### Governing equation

We denote an imaging object as *Ω*. The admittivity in *Ω* is *κ*=*σ*(*ω*)+*iωε*(*ω*), where *σ*(*ω*) and *ε*(*ω*) are the conductivity and permittivity, respectively, at the angular frequency *ω*. Time-harmonic Maxwell’s equations relate the electric field **E** and magnetic flux field **H**:

(1) $$\nabla \times E(r)=-\mathrm{i\omega}\mu_{0}H(r)\;\text{and}\;\nabla \times H(r)=\kappa (r)E(r),\;r\in \Omega ,$$

where the current **J** and the electric field intensity **E** satisfy the relation **J**=*κ***E** by Ohm’s law.

In MREIT, we can assume that the injecting current is sufficiently low frequency to meet *iωε*(*ω*)≈0 and neglect *iωμ*_0_≈0, Maxwell’s equation (1) satisfies

(2) $$\begin{array}{ll} -\nabla^{2}H(r) & =\nabla \times \nabla \times H(r)=\nabla \times (\sigma_{L}(r)E(r))\\ & =\nabla \sigma_{L}(r)\times E(r)=\frac{\nabla \sigma_{L}(r)}{\sigma_{L}(r)}\times (\nabla \times H(r)),\;r\in \mathrm{\Omega .} \end{array}$$

where *σ*_*L*_=*σ*(*ω*) denotes the low-frequency electrical conductivity responding to the externally injecting current. Since the electric field intensity **E**=−∇*u* is a gradient form under negligible angular frequency *ω*, we have

(3) $$\left\{\begin{array}{cccc} \nabla \cdot (\sigma_{L}\nabla u(r)) & = & 0\; & \text{in}\;\;\Omega \\ -\sigma_{L}\nabla u(r)\cdot n(r) & = & g(r)\; & \text{on}\;\partial \Omega \end{array}\right)$$

where **n** is the outward normal vector on the surface *∂Ω* and *g* is the current density on the surface.

In MREPT, by assuming the locally homogeneity, the magnetic field **H** can be expressed as a simple form:

(4) $$-\nabla^{2}H(r)=\nabla \kappa (r)\times E(r)-\mathrm{i\omega}\mu_{0}\kappa (r)H(r)=-\mathrm{i\omega}\mu_{0}\kappa (r)H(r),\;r\in \mathrm{\Omega .}$$

The phase terms retrieved from the measured phase are the combination of the phase terms of positive $H^{+}=\frac{H_{x}+{\text{iH}}_{y}}{2}$ and negative $H^{-}=\frac{H_{x}-{\text{iH}}_{y}}{2}$ rotating field. For a restricted situation such as the usage of transmit-receive coil, the phase term of positive rotating field can be retrieved from the measured phase. The transverse field of **H** can be decomposed into the positively rotating field $H^{+}=\frac{H_{x}+{\text{iH}}_{y}}{2}$ and the negatively rotating field $H^{-}=\frac{H_{x}-{\text{iH}}_{y}}{2}$. We denote *φ*^+^ and *φ*^−^ as the phase term of *H*^+^ and *H*^−^, respectively. When single channel transmit-receive coils are used, the transmit *φ*^+^ can be reasonably similar to *φ*^−^[16]. The measurable positively rotating field $H^{+}=\frac{H_{x}+{\text{iH}}_{y}}{2}$ of **H**=(*H*_*x*_,*H*_*y*_,*H*_*z*_) also satisfies the relation under the locally homogeneity condition

(5) $$-\nabla^{2}H^{+}(r)=-\mathrm{i\omega}\mu_{0}\kappa (r)H^{+}(r),\;r\in \mathrm{\Omega .}$$

By separating the real and imaginary parts of *κ* and assuming *φ*^+^≈*φ*^−^, the phase term of *H*^+^ can estimate the conductivity *σ*_*H*_=*σ*(*ω*) which reflects the high-frequency property responding to the RF pulse [16]:

(6) $$\sigma_{H}(r)\approx \frac{\nabla^{2}\varphi^{+}(r)}{\mu_{0}\omega },\;r\in \mathrm{\Omega .}$$

### ICNE multi-echo pulse sequence and multiple phase data

**Figure 1:**
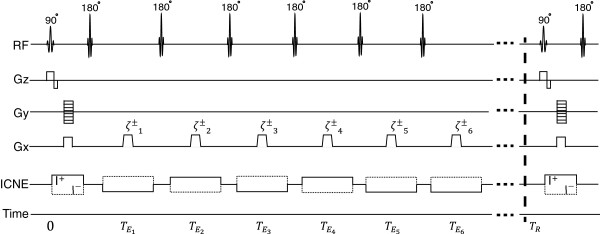
**Schematic diagram for ICNE multi-echo pulse sequence to acquire dual-frequency conductivity images.** ‘RF’ shows the timing for excitation of 90° and 180° RF pulses. *G*_*z*_, *G*_*y*_ and *G*_*x*_ are slice selection gradient, phase encoding gradient and readout gradient, respectively. $\zeta_{j}^{\pm }$ is the *j*-th echo signal. ‘ICNE’ shows the alternating injection currents in the form of pulses synchronized with the RF pulse.

The ICNE multi-echo pulse sequence is adopted to acquire multiple echoes measured at the echo time $T_{E_{j}},\;j=1,\cdots \;,N_{E}$, where *N*_*E*_ is the number of echoes per repetition time *T*_*R*_. Figure 1 shows its schematic diagram where *I*^+^ and *I*^−^ are sequentially injected positive and negative currents synchronized with the applied 180° rephasing pulses with *N*_*E*_ times. The *j*-th complex image, $\zeta_{j}^{\pm }$, corresponding to the *j*-th readout gradient generated with the positive and negative injecting currents *I*^+^ and *I*^−^, can be expressed as

(7) $$\zeta_{j}^{\pm }(r)=M_{\text{xy},j}^{\alpha }(r)e^{i\delta_{\varepsilon }}e^{i\varphi^{+}}e^{\pm {\left(-1\right)}^{\;j}\mathrm{i\gamma}T_{c^{\;j}}B_{z^{\;j}}(r)},\;j=1,\cdots \;,N_{E}$$

where $M_{\text{xy},j}^{\alpha }(r)=M_{\text{xy}}^{\alpha }(r)e^{-T_{E_{j}}/T_{2}(r)}$ is the *j*-th transverse magnetization at a flip angle *α*, *δ*_*ε*_ is the systematic phase artifact, *γ* is the gyromagnetic ratio of hydrogen, *T*_2_ is the transverse relaxation time, and $T_{c^{\;j}}$ is the *j*-th duration of the injecting current.

The *j*-th transverse magnetization $M_{\text{xy},j}^{\alpha }(r)$ can be rewritten in detail as

(8) $$M_{\text{xy},j}^{\alpha }(r)=C_{1}M_{0}(r)H^{-}(r)\sin(C_{2}\alpha |H^{+}(r)|)e^{-\frac{T_{E_{j}}}{T_{2}(r)}},\;j=1,\cdots \;,N_{E}$$

where *C*_1_ and *C*_2_ are system-dependent constants. Assuming *δ*_*ε*_≈0, the phase signal can be separated as

(9) $$P_{j}^{\pm }(r):=\text{arg}\left(\zeta_{j}^{\pm }(r)\right)=2\varphi^{+}(r)\pm {\left(-1\right)}^{\;j}\gamma T_{c^{\;j}}B_{z^{\;j}}(r),\;j=1,\cdots \;,N_{E}\;.$$

By subtracting and adding the phase signals $P_{j}^{\pm }$, we can obtain the magnetic flux density *B*_*z*_ induced by the externally injecting current at the low-frequency and the phase term of *H*^+^ at the Larmor frequency simultaneously.

(10) $$\begin{array}{cccc} P_{j}^{+}(r)+P_{j}^{-}(r) & = & 4\varphi_{j}^{+}(r), & \;j=1,\cdots \;,N_{E}\\ P_{j}^{+}(r)-P_{j}^{-}(r) & = & 2\gamma T_{c^{\;j}}B_{z^{\;j}}(r), & \;j=1,\cdots \;,N_{E} \end{array}$$

From the relation 10, we can compute the *j*-th phase signals for MREPT and the *j*-th *z*-component of magnetic flux density for MREIT:

(11) $$\begin{array}{cccc} \varphi_{j}^{+} & = & \frac{P_{j}^{+}+P_{j}^{-}}{4}, & \;j=1,\cdots \;,N_{E}\\ B_{z^{\;j}} & = & \frac{P_{j}^{+}-P_{j}^{-}}{2\gamma T_{c^{\;j}}}, & \;j=1,\cdots \;,N_{E} \end{array}$$

The noise standard deviation of the measured magnetic flux density $B_{z^{\;j}}$ in 11 is inversely proportional to the current injection time $T_{c}^{\;j}$ and the signal-to-noise ratio (*Υ*^*j*^) of MR magnitude image following [27,28]

(12) $${\text{sd}}_{B_{z^{\;j}}}(r)=\frac{1}{2\gamma T_{c}^{\;j}\Upsilon^{j}(r)},\;j=1,\cdots \;,N_{E}\;.$$

A similar analysis provides the noise standard deviation of *φj*+ as

(13) $${\text{sd}}_{\varphi_{j}^{+}}(r)=\frac{1}{4\Upsilon^{j}(r)},\;j=1,\cdots \;,N_{E}\;.$$

### Optimal combination of multiple $B_{z^{\;j}}$

In order to enhance the conductivity image at the low frequency using MREIT, we have to improve the quality of the *B*_*z*_ signal. An alternative to reduce the noise of *B*_*z*_ is to find an optimal combination of multiple $B_{z^{\;j}},\;j=1,\cdots \;,N_{E},$ by determining the weighting factors which satisfied conditions of *ξ*_*j*_(**r**)>0 and $\overset{N_{E}}{\underset{j=1}{\sum }}\xi_{j}(r)=1$. When we assume that the measured *k*-space signals are contaminated with independent identically distributed (IID) complex Gaussian random noise as same amount of expected noise standard deviation of each $B_{z^{\;j}}$ in 12, the noise variance of the weighted $B_{z^{\;j}}$ satisfies the following relation

(14) $$\text{Var}(B_{z}^{\xi }(r))=\overset{N_{E}}{\underset{j=1}{\sum }}\xi_{j}^{2}(r)\text{Var}(B_{z^{\;j}}(r))\propto \overset{N_{E}}{\underset{j=1}{\sum }}\frac{\xi_{j}^{2}(r)}{T_{c^{\;j}}^{2}|\zeta_{j}^{\pm }(r){|}^{2}}\;.$$

To determine the optimized weighting factor *ξ*_*j*_(**r**), the noise variance of the combined $B_{z}^{\xi }$ is required to minimize the following conditions:

(15) $$\left\{\begin{array}{l} \underset{\xi_{j},j=1,\cdots \;,N_{E}}{\min}\;F_{B_{z}}(\xi_{1},\cdots \;,\xi_{N_{E}})(r)\\ \text{subject to}\;G_{B_{z}}(\xi_{1},\cdots \;,\xi_{N_{E}})(r)=1,\;\xi_{j}(r)>0 \end{array}\right)$$

where $F_{B_{z}}(\xi_{1},\cdots \;,\xi_{N_{E}}):=\overset{N_{E}}{\underset{j=1}{\sum }}\frac{\xi_{j}^{2}(r)}{T_{c^{\;j}}^{2}{\left|\zeta_{j}^{\pm }(r)\right|}^{2}}$ and $G_{B_{z}}(\xi_{1},\cdots \;,\xi_{N_{E}}):=\overset{N_{E}}{\underset{j=1}{\sum }}\xi_{j}(r)$. The method of Lagrange multipliers to solve the optimization problem in 15 introduces a new variable *λ* and requires the condition that the gradients of $F_{B_{z}}$ and $G_{B_{z}}$ are parallel. Using the method of Lagrange multipliers, the optimal weighting factor *ξ*_*j*_(**r**) can be obtained as

(16) $$\xi_{j}(r)=\frac{\Psi_{j}(r)}{\overset{N_{E}}{\underset{j=1}{\sum }}\Psi_{j}(r)}$$

where $\Psi_{j}(r):=T_{c^{\;j}}^{2}|\zeta_{j}^{\pm }(r){|}^{2}$.

The optimized magnetic flux density to produce a conductivity image in MREIT can be generated by the weighted average of multiple $B_{z^{\;j}}$ with the determined weighting factor of *ξ*_*j*_(**r**) as $B_{z}^{\xi }(r)=\overset{N_{E}}{\underset{j=1}{\sum }}\xi_{j}(r)B_{z^{\;j}}(r)$ using the ICNE multi-echo pulse sequence. The noise standard deviation of $B_{z}^{\xi }$ can be precisely described by substituting 16 into the noise variance of $B_{z}^{\xi }$ in 14:

(17) $${\text{sd}}_{B_{z}^{\xi }}(r)=\frac{N_{M_{\text{xy}}}(r)}{2\gamma M_{\text{xy}}^{\alpha }(r)\sqrt{\overset{N_{E}}{\underset{j=1}{\sum }}T_{c^{\;j}}^{2}e^{-\frac{2T_{E_{j}}}{T_{2}(r)}}}}\;.$$

where $N_{M_{\text{xy}}}$ denotes the noise level of magnitude image.

### Optimal combination of multiple *φj*+

The noise variance of the weighted *φj*+ satisfies the following relation similar as 14.

(18) $$\text{Var}(\varphi^{+,\chi })=\overset{N_{E}}{\underset{j=1}{\sum }}\chi_{j}^{2}(r)\text{Var}(\varphi_{j}^{+})\propto \overset{N_{E}}{\underset{j=1}{\sum }}\frac{\chi_{j}^{2}(r)}{|\zeta_{j}^{\pm }(r){|}^{2}}$$

The noise variance of the combined *φ*^+^ with weighting factors is required to minimize the following conditions,

(19) $$\left\{\begin{array}{l} \underset{\chi_{j},j=1,\cdots \;,N_{E}}{\min}\;F_{\varphi^{+}}(\chi_{1},\cdots \;,\chi_{N_{E}})(r)\\ \text{subject to}\;G_{\varphi^{+}}(\chi_{1},\cdots \;,\chi_{N_{E}})(r)=1,\;\chi_{j}(r)>0 \end{array}\right)$$

where $F_{\varphi^{+}}(\chi_{1},\cdots \;,\chi_{N_{E}}):=\overset{N_{E}}{\underset{j=1}{\sum }}\frac{\chi_{j}^{2}(r)}{|\zeta_{j}^{\pm }(r){|}^{2}}$ and $G_{\varphi^{+}}(\chi_{1},\cdots \;,\chi_{N_{E}}):=\overset{N_{E}}{\underset{j=1}{\sum }}\chi_{j}(r)$. The optimal weighting factor, *χ*_*j*_(**r**), corresponding to each echo phase can be determined by using the method of the Lagrange multipliers as

(20) $$\chi_{j}(r)=\frac{\Phi_{j}(r)}{\overset{N_{E}}{\underset{j=1}{\sum }}\Phi_{j}(r)}$$

where $\Phi_{j}(r):=|\zeta_{j}^{\pm }(r){|}^{2}$.

The optimally weighted average of multiple phase signal *φj*+ with the determined weighting factor of *χ*_*j*_(**r**), $\varphi^{+,\chi }(r)=\overset{N_{E}}{\underset{j=1}{\sum }}\chi_{j}(r)\varphi_{j}^{+}(r)$, can produce an enhanced conductivity image in MREPT. The noise standard deviation of the optimized phase signal, *φ*^+,*χ*^, is computed by substituting (20) into the noise variance of *φ*^+,*χ*^ in (18) to estimate pixel-by-pixel noise precisely:

(21) $${\text{sd}}_{\varphi^{+,\chi }}(r)=\frac{N_{M_{\text{xy}}}(r)}{4M_{\text{xy}}^{\alpha }(r)\sqrt{\overset{N_{E}}{\underset{j=1}{\sum }}e^{-\frac{2T_{E_{j}}}{T_{2}(r)}}}}$$

Considering only random noise effects, the optimal weighting factor *χ*_*j*_(**r**) reduces the noise level, depending on the echo number and *T*_2_-decay rate at each imaging pixel.

### Denoising method using the estimated ${\text{sd}}_{B_{z}^{\xi }}$ and ${\text{sd}}_{\varphi^{+,\chi }}$

A denoising technique is applied to gradients of the optimized magnetic flux density for MREIT image and the optimized phase signal for MREPT image since the reconstruction procedures only require the differentiated measured data. Also, it is advantageous to denoise $\nabla B_{z}^{\xi }$ and ∇*φ*^+,*χ*^ because the measured magnetic flux density of $B_{z}^{\xi }$ and the phase of *H*^+^ are inherently continuous without conventional edge information.

To denoise the optimized $\nabla B_{z}^{\xi }$ and ∇*φ*^+,*χ*^, we use the following conventional reaction-diffusion equation [29,30]:

(22) $$\left\{\begin{array}{lll} \frac{\mathrm{\partial v}}{\mathrm{\partial t}}(r,t) & = & \nabla \cdot \left(\psi \left(r\right)\tilde{\nabla }v(r,t)\right)-\beta (r)(v(r,t)-f(r))\\ v(r,0) & = & f(r) \end{array}\right)$$

where the parameter *β* is a fidelity term and the function *ψ* satisfies

(23) $$\psi (s)>0,\;\underset{s\to \infty }{\lim}\psi (s)=0\;.$$

Here, the initial state *f* denotes each component of the optimized magnetic flux density $\nabla B_{z}^{\xi }$ or the phase signal ∇*φ*^+,*χ*^, which is to be denoised. The time dependent solution *v*(**r**,*t*) is the denoised image of the initial state image *f*.

To remove the random noise artifact while preserving the edge information, it is important to determine the diffusion function *ψ* and *β*. Using the estimated noise level of ${\text{sd}}_{\varphi^{+,\chi }}$, the diffusion function *ψ* and the fidelity function *β* can be specified as

(24) $$\psi (r)\propto \frac{1}{|\nabla v(r,t)|+\varsigma_{\varepsilon }}\;\text{and}\;\beta (r)\propto {\text{sd}}_{\varphi^{+,\chi }}(r)$$

where *ς*_*ε*_ is a small parameter to guarantee the positive sign of |∇*v*(**r**,*t*)|+*ς*_*ε*_. For the magnetic flux density $B_{z}^{\xi }$, the diffusion function *ψ* can be selected similarly.

The quality of magnetic flux density signal in MREIT depends on the magnitude intensity and width of injected current simultaneously, while the quality of phase signal in MREPT only depends on the magnitude intensity. Therefore, there are common effects due to the decay of the magnitude intensity in MREPT and MREIT processing. There are also different effects caused by externally injecting current in MREIT. These occur when we calculate each of the optimal weighting factors and the noise standard deviation in the optimal combination of multiple phase and magnetic flux density images. The proposed optimization method uses the decay rate of magnitude intensities to determine the weighting factors in MREIT and MREPT, which relate with the *T*_2_ values. However, the determined weighting factors in 16 and 20 only include the measured magnitude intensity at each echo time $T_{E_{j}},\;j=1,\cdots \;,N_{E}$. The proposed method therefore does not need estimates *T*_2_ values to optimize the multiple phase signals.

### Phantom design and experimental setup

**Figure 2:**
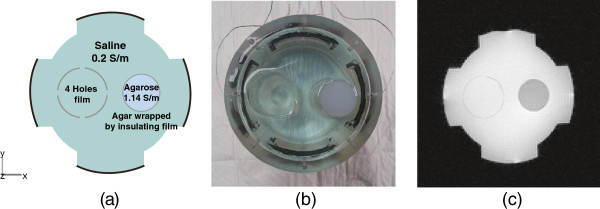
**Experimental set-up for a phantom.(a)** The phantom configuration at the middle slice of the testing object, **(b)** a cylindrical phantom with saline solution of 0.2 *Sm*^−1^ including a thin film object and an agarose anomaly wrapped by an insulating film, and **(c)** a magnitude image at the middle slice acquired at *T*_*E*_=15 ms.

In order to evaluate the proposed optimizing method using ICNE multi-echo MR pulse sequence, we designed a phantom which can show different characteristics of reconstructed conductivity images by MREIT and MREPT. Figure 2(a) and (b) show the phantom configuration at the middle slice of the object and the photo view of the phantom, respectively. We built a cylindrical phantom filled with a saline of 0.2 *Sm*^−1^ conductivity (0.3 g/L NaCl and 1 g/L CuSO_4_). The diameter and height of the phantom were 11 cm and 14 cm, respectively. We attached four carbon hydrogel electrodes (HUREV Co. Ltd, Korea) on the side of the acrylic container for injecting current.

Two different objects were positioned inside phantom. The left one was a thin hollow cylindrical object with the diameter of 4 cm using an insulating thin film of 0.4 mm thickness. We punched four holes which had 2 mm diameter with equally spaced around the circumference. Because we filled the same saline of 0.2 *Sm*^−1^ inside and outside of the hollow cylindrical object with holes, the apparent electrical conductivities inside and outside of object were determined by the movement of ions through the holes [31]. The right cylindrical object with the diameter of 3 cm was made of an agarose gel (1 g/L CuSO_4_, 2.1 g/L NaCl, 15 g/L Agar) to generate different conductivity, spin density and *T*_2_-decay compared with the background saline. The conductivity of agarose gel was 1.10 *Sm*^−1^. It was wrapped in a thin insulating film without holes.

A transversal injecting current of 10 mA was introduced into the phantom *via* a pair of recessed carbon hydrogel electrodes attached at the middle of the phantom. Figure 2(c) shows the magnitude image at the middle slice. We used the ICNE multi-echo MR pulse sequence by injecting current of alternating polarity synchronized with the multiple refocusing pulses as shown in Figure 1.

Imaging parameters in a 3T MRI scanner (Achieva, Philips) with birdcage transmit-receive (Tx/Rx) RF head coil were as follows: repetition time *T*_*R*_=1200 ms, data acquisition time width *T*_*s*_=3.584 ms, echo-spacing $T_{E_{\text{sp}}}=15$ ms, total number of echo *N*_*E*_=6 and number of averaging = 4. The reconstructed image matrix was 128×128, with a FOV of 180×180 mm^2^, 5 mm slice thickness. Total scanning time for both vertical and horizontal injection currents was 20 minutes with an interleaved phase encoding acquisition.

## Results

**Figure 3:**
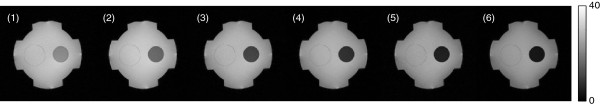
**MR magnitude images measured at each echo time.** Echo number marked on the upper left part of each image as (1), (2), ⋯, (6). Magnitude images using ICNE multi-echo pulse sequence acquired at the echo time $T_{E_{j}}=15\times j$ ms for *j*=1,⋯,6.

Figure 3 shows the multiple MR magnitude images measured at the echo time $T_{E_{j}}=15\times j$ ms for *j*=1,⋯,6, where the right anomaly shows rapid decay of the transverse magnetization due to magnetic field non-uniformity and spin-spin transverse relaxation.

**Figure 4:**
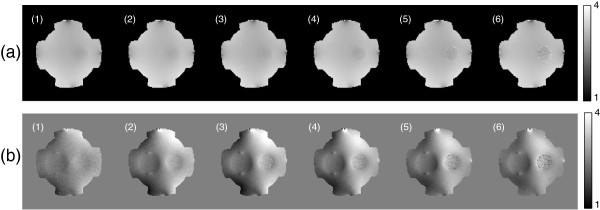
**Phase images and magnetic flux density images of each echo.(a)** Phase images *φj*+ of *H*^+^ map in radian and **(b)** magnetic flux density images $B_{z^{\;j}}$ in ’nT’ unit using ICNE multi-echo pulse sequence at the echo time $T_{E_{j}}=15\times j$ ms for *j*=1,⋯,6.

Figure 4 shows the phase images of *φj*+ of *H*^+^ map and magnetic flux density $B_{z^{\;j}}$ induced by the vertically injected current using ICNE multi-echo pulse sequence at the echo time $T_{E_{j}}=15\times j$ ms, *j*=1,⋯,6. The images in Figure 4(a) and (b) were estimated by adding and subtracting the phase signals of $\zeta_{j}^{\pm }$, respectively. Since the electromagnetic wave at the Larmor frequency of 128 MHz could penetrate the thin insulating film and there was same saline inside and outside of the thin insulating wall of the left hollow cylinder, the measured phase values of *φj*+ for the left object in MREPT could not produce conductivity difference between inside and outside. However, the measured $B_{z^{\;j}}$ images at the low frequency in MREIT showed the different value inside the left anomaly because MREIT measures the apparent magnetic flux density corresponding to the ion mobility and intrinsic conductivities of composite materials. Note that the slope of magnetic flux density was changed abruptly due to the high current density around the holes in the same direction of the current injection. Regarding the different conductivity agarose gel cylinder wrapped in the thin insulating film without holes, both images in Figure 4(a) and (b) could present distinctions between inside of the object and the background saline.

**Figure 5:**
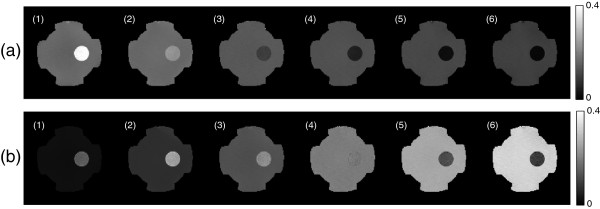
**Images of weighting factors corresponding to each echo.(a)** Images of weighting factors corresponding to each echoes for *φj*+ and **(b)** images for magnetic flux density $B_{z^{\;j}}$ to produce an optimal *φ*^+,*χ*^ and $B_{z}^{\xi }$, respectively. The weighting factors were scaled from 0 to 1.

Using the proposed optimization method with the estimated noise standard deviations ${\text{sd}}_{\varphi_{j}^{+}}$ and ${\text{sd}}_{B_{z}^{\;j}},\;j=1,\cdots \;,6$, we found the optimal weighting factors for the phase of *H*^+^ and the magnetic flux density by the injected current, respectively. Figure 5(a) and (b) show the determined weighting factors for dual-frequency conductivity images in MREPT and MREIT combined method, respectively. In MREPT, the intensity of weighting factors was monotonically decreased because the noise level of *φj*+ only depended on *T*_2_-decay rate of $\zeta_{j}^{\pm },\;j=1,\cdots \;,6$, whereas the weighting factors in MREIT show the different characteristics because they depended on the combination of *T*_2_-decay rate and the duration of injected current simultaneously. In Figure 5(b), the intensity of weighting factors in the background area was monotonically increased because the effect of time increasing for the injection current was greater than the magnitude attenuation effect of echo signals. However, the weighting factors in the right agarose gel anomaly region were different due to the combination of both effects. The weighting factor values at the second echo were higher than those of other echoes.

**Figure 6:**
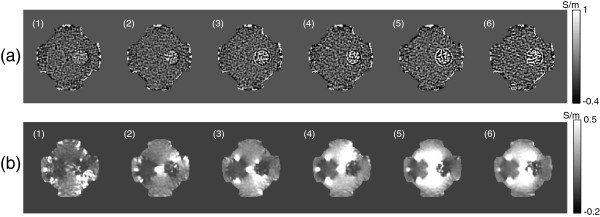
**Reconstructed conductivity images of each echo.(a)** Reconstructed conductivity images using the multiple echoes *φj*+ for *j*=1,⋯,6 at Larmor frequency and **(b)** conductivity images using $B_{z^{\;j}}^{d}$ for *j*=1,⋯,6 at the low frequency.

Figure 6 shows the reconstructed dual-frequency conductivity images using the multiple echoes *φj*+ and $B_{z^{\;j}}^{d}$ for *j*=1,⋯,6 and *d*=1,2, where $B_{z^{\;j}}^{1}$ and $B_{z^{\;j}}^{2}$ were the measured magnetic flux density data by the vertically and horizontally injected currents, respectively. To reconstruct the high-frequency conductivity distribution in Figure 6(a), we used phase for each echo signal as

(25) $$\sigma_{H}^{j}(r)=\frac{\nabla^{2}\varphi_{j}^{+}(r)}{\mu_{0}\omega },\;r\in \Omega ,\;j=1,\cdots \;,6\;.$$

Here, we could not detect the hollow cylinder in the left side made of the thin insulating film with holes. The high-frequency conductivity inside and outside of the left object was estimated to be the same value. The quality of reconstructed conductivity image for each echo in Figure 6(a) was monotonically degraded corresponding to the weighting factors shown in Figure 5(a). Table 1 shows the values of weighting factor inside and outside of the right agarose object wrapped by insulating film.

**Table 1:** **Weighting factor values for MREPT inside (**$R_{\text{in}}$**) and outside (**$R_{\text{out}}$**) the agarose anomaly**

	***N***_***E***_ ***=1***	***N***_***E***_ ***=2***	***N***_***E***_ ***=3***	***N***_***E***_ ***=4***	***N***_***E***_ ***=5***	***N***_***E***_ ***=6***
$R_{\text{out}}$	0.2030	0.2046	0.1737	0.1570	0.1385	0.1232
$R_{\text{in}}$	0.4926	0.2830	0.1215	0.0617	0.0268	0.0143

We estimated the projected current density **J**^*P*^, which was the optimal divergence-free component from the measured *z*-component magnetic flux density in order to reconstruct the low-frequency conductivity images [32]. The projected current density consists of the background current density **J**^**0**^=−∇*α* and$\nabla_{\text{xy}}^{⊥}\eta =\left(\frac{\partial \eta }{\mathrm{\partial y}},-\frac{\partial \eta }{\mathrm{\partial x}},0\right)$, where the potential *η* satisfies at the imaging slice *Ω*_*t*_⊂*Ω*

(26) $$\begin{array}{cccc} \nabla_{\text{xy}}^{2}\eta & = & \frac{1}{\mu_{0}}\nabla^{2}B_{z}\; & \text{in}\Omega_{t}\\ \eta & = & 0 & \text{on}\;\partial \Omega_{t}. \end{array}$$

where ∇_*xy*_denotes the two-dimensional gradient. The absolute conductivity distribution can be written as a matrix form using the two measured$B_{z}^{d},\;d=1,2,$data

(27) $$\left(A_{0}+A^{*}\right){\left(\frac{\partial \tau }{\mathrm{\partial x}},\frac{\partial \tau }{\mathrm{\partial y}}\right)}^{T}=b\;\;\text{in}\;\Omega_{t}$$

where *Ω*_*t*_is an imaging slice, *τ*= log*σ*, and

(28) $$A_{0}=\left(\begin{array}{cc} \frac{-\partial \alpha^{1}}{\mathrm{\partial y}} & \frac{\partial \alpha^{1}}{\mathrm{\partial x}}\\ \frac{-\partial \alpha^{2}}{\mathrm{\partial y}} & \frac{\partial \alpha^{2}}{\mathrm{\partial x}} \end{array}\right),\;A^{*}=\left(\begin{array}{cc} \frac{\partial \eta^{1}}{\mathrm{\partial x}} & -\frac{\partial \eta^{1}}{\mathrm{\partial y}}\\ \frac{\partial \eta^{2}}{\mathrm{\partial x}} & -\frac{\partial \eta^{2}}{\mathrm{\partial y}} \end{array}\right),\;b=\frac{1}{\mu_{0}}\left(\begin{array}{c} \nabla^{2}B_{z}^{1}\\ \nabla^{2}B_{z}^{2} \end{array}\right)\;.$$

Figure 6(b) shows the reconstructed low-frequency conductivity images by solving 27 using the *j*-th measured two magnetic flux densities$B_{z^{\;j}}^{d},\;d=1,2$obtained with the vertically and horizontally injected currents. The reconstructed conductivity values inside the hollow cylinder with holes in the left side reflected the externally injected current flowed through the four holes. Regarding the agarose gel object wrapped in the insulating film without holes, the apparent conductivity reconstructed by solving 27 shows the same anomaly as an insulator since the measured *B*_*z*_data only reflected the current density distribution due to the externally injected current through the attached electrodes. The quality of conductivity image using the sixth echo data was poor inside the agarose gel anomaly region due to relatively short *T*_2_-decay relaxation time. It was consistent with the corresponding weighting factor in Figure 5(b). Table 2 shows the values of weighting factor inside and outside of the right agarose object wrapped by insulating film.

**Table 2:** **Weighting factor values for MREIT inside (**$R_{\mathrm{in}}$**) and outside (**$R_{\mathrm{out}}$**) the agarose anomaly**

	***N***_***E***_ ***=1***	***N***_***E***_ ***=2***	***N***_***E***_ ***=3***	***N***_***E***_ ***=4***	***N***_***E***_ ***=5***	***N***_***E***_ ***=6***
$R_{\text{out}}$	0.0238	0.0734	0.1269	0.1936	0.2585	0.3239
$R_{\text{in}}$	0.1404	0.2466	0.2158	0.1849	0.1214	0.0910

**Figure 7:**
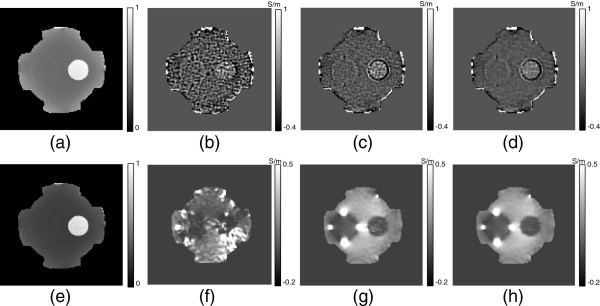
**Optimized dual-frequency conductivity images with and without applying the optimization and denoising method.(a)** and **(e)** Normalized noise standard deviation of the optimally weighted phase of *H*^+^and$B_{z}^{\xi }$, respectively. **(b)** and **(f)** Reconstructed conductivity images without using the optimization and denoising method. **(c)** and **(d)** Reconstructed conductivity distributions at Larmor frequency using the optimized *φ*^+,*χ*^without denoising and with the proposed denoising technique applying to ∇*φ*^+,*χ*^, respectively. **(g)** and **(h)** Reconstructed low-frequency conductivity distributions without denoising and with the proposed denoising technique applying to$\nabla B_{z}^{d,\xi },\;d=1,2$, respectively.

Figure 7(a) and (e) show the normalized noise standard deviations of optimally weighted *φ*^+,*χ*^and$B_{z}^{\xi }$, respectively. In previous approaches without using multi-echo data with optimizing method, the conductivity images for MREPT and MREIT were reconstructed as shown in Figure 7(b) and (f). Due to the different *T*_2_-decay rate and the duration of injected current, the intensities of${\text{sd}}_{\varphi^{+,\chi }}$and${\text{sd}}_{B_{z}^{\xi }}$represented different characteristics. The reconstructed dual-frequency conductivity images with the optimally weighted *φ*^+,*χ*^and$B_{z}^{d,\xi },\;d=1,2,$were displayed in Figure 7(c) and (g). Solving the reaction-diffusion equation 22 with the estimated noise standard deviations${\text{sd}}_{\varphi^{+,\chi }}$and${\text{sd}}_{B_{z}^{\xi }}$, we denoised ∇*φ*^+,*χ*^and$\nabla B_{z}^{d,\xi }$; the iteration number was 400 and the regularization function of *β* was$\frac{0.05}{N({\text{sd}}_{\varphi^{+,\chi }})}$for MREPT case, where$N({\text{sd}}_{\varphi^{+,\chi }})$was the normalized noise standard deviation in Figure 7(a). The denoising parameters for MREIT were the same as those for MREPT. Figure 7(d) and (h) show the reconstructed dual-frequency conductivity images after denoising of ∇*φ*^+,*χ*^and$\nabla B_{z}^{d,\xi }$, respectively.

## Discussion

MREPT and MREIT used an internal magnetic flux density information from a phase imaging can provide conductivity images with high resolution compared to the conventional electrical impedance imaging techniques. Moreover, the interleaved measurement technique with alternating injected current polarity based on a multi-spin-echo pulse sequence, enables simultaneous dual conductivity images. From a single MR scan, we could obtain the low-frequency conductivity images by subtracting and the high-frequency conductivity images by adding the measured phase terms. Despite their advantages and usefulness, MREPT and MREIT techniques commonly suffer from noise amplifications in phase imaging. The proposed method used a ICNE multi-echo pulse sequence based on a spin echo pulse sequence in order to suppress the background field inhomogeneity. This also influenced to the reconstructed high-frequency conductivity using the MREPT technique. Even though the estimated weighting factors for MREIT and MREPT optimally reduce the random noise artifacts, non-uniform noise artifacts from various sources can severely deteriorate the reconstructed conductivity distributions. To be a clinical device used practically, it is important to develop noise reduction techniques taking into account the physical properties and experimental environments of MREIT and MREPT.

The designed phantom consists of two different kinds of anomalies to show the frequency dependent conductivity information. The stable apparent conductivity contrast of hollow cylindrical object made of thin insulating transparency film was only controlled by ion mobility through holes on the film wall. It excluded other effects caused by any ion concentration gradient. Therefore, MREIT images could only present the hollow cylindrical object with holes when comparing the phase terms$P^{+}$and$P^{-}$in 10. The apparent conductivity of the region inside cylinder was a non-zero conductivity value because the externally injected current entered the anomaly through the holes. In MREPT, the reconstructed high-frequency conductivity was same inside and outside of the anomaly since the measured phase of *H*^+^only reflected the material conductivities inside and outside of the cylindrical film. Another agarose gel cylinder wrapped in an insulating film without holes shows different conductivity characteristics in MREPT and MREIT. Due to the short *T*_2_-decay relaxation time of agarose gel, the noise levels of${\text{sd}}_{\varphi^{+,\chi }}$and${\text{sd}}_{B_{z}^{\xi }}$were relatively severe compared to those in the other regions. The noise level of each${\text{sd}}_{\varphi_{j}^{+}}$was strictly increasing with respect to the echo number depending on the *T*_2_-decay rate, whereas those of${\text{sd}}_{B_{z^{\;j}}}$had different characteristics depending on$T_{c^{\;j}}$and the *T*_2_-decay rate simultaneously.

MREIT provides the conductivity distribution at a low frequency, whereas MREPT produces the conductivity at the Larmor frequency of 128 MHz at 3 T. The cell membranes consisted of the phospholipid bilayer with embedded proteins behave as a capacitor or insulating film. It shows a complicated pattern of conductivity depending on the measured frequencies. For the biological tissues, the membranes restrict the flow of current of low frequency. MREIT images reflect the membrane properties quantitatively and MREIT has a potential to visualize the anisotropic conductivity tensor map. On the other hand, the membranes become transparent at high frequency and provide a relatively degraded sensitivity in the conductivity image. MREPT may provide different conductivity characteristics compared to MREIT results. Based on the previous studies related to the complex conductivity spectra measured at multiple frequencies within the range of 10 Hz to several kHz, spectroscopic complex conductivity distribution can contribute to explain the physiological and pathological status of internal tissues [33]. The dual-frequency conductivity imaging by combination of MREPT and MREIT is an advanced technique to show different information in spite of displaying two extreme cases at low and the Larmor frequencies. It is meaningful to develop a method to produce multi-frequency conductivity image spectra with high spatial resolution and sensitivity using MR scanner within the range of DC to the Larmor frequency since significantly distinguishable signal changes in biological tissues depending on the applied frequency range.

We have a plan to support the clinically applicable combined conductivity imaging method based on MR techniques. We may apply this method to detect the destroyed cell membrane or the modification of cell or tissue structure due to necrosis or apoptosis initiated by inflammatory response inside the body. Since RF ablation and cryoablation for cancer treatments are accompanied by the destruction of cell membranes, it will be a good method to diagnose ablated lesions based on physiological status of tissue to improve the safety and predict the local recurrence after ablation.

## Conclusions

Cross-sectional conductivity imaging methods for high spatial resolution and sensitivity inside the human body has been actively investigated. MREIT and MREPT techniques show different electrical properties at low frequency (below 1 kHz) as MREIT uses an externally injecting current while MREPT uses the Larmor frequency of an MRI scanner. Recently, the dual-frequency conductivity imaging from a single MR scan simultaneously was proposed based on combination of MREPT and MREIT. Even though it produces conductivity spectral information, MREPT and MREIT have commonly suffered from weak signals and noise amplification since the procedures to reconstruct the conductivity in MREIT and MREPT need to differentiate the measured phase signals. We suggested the optimization method to find weighting factors according to echo signals for MREPT and MREIT using the ICNE multi-echo pulse sequence, which minimized the noise artifacts in the measured phase data. The noise standard deviations were precisely analyzed for the optimally weighted magnetic flux density and the phase term of positive-rotating magnetic field. We applied the denoising method based on the reaction-diffusion equation with the estimated noise standard deviations to enhance the quality of dual-frequency conductivity images. A real phantom experiment was performed to validate the proposed method using common measured data to reconstruct the dual-frequency conductivity distributions for MREPT and MREIT.

## Competing interests

The authors declare that they have no competing interests.

## Authors’ contributions

OIK developed the algorithm and wrote the manuscript. WCJ prepared and performed the experiments and wrote up the experimental section. SZKS analyzed the experimental data and confirmed it by simulation. HJK assisted with analysis and processing the data. EJW conceived the idea and gave critical revision for important intellectual content. TIO designed the research topic and experiments, analyzed and drafted the manuscript. All authors read and approved the final manuscript.
